# Factors associated with recurrence of mitral regurgitation 14 days after rheumatic mitral valve repair

**DOI:** 10.3389/fcvm.2026.1797235

**Published:** 2026-04-28

**Authors:** RenDong Liang, Ding Zhang, QingYu Sun, BiJun Zhao, Xu Meng

**Affiliations:** 1 Department of Cardiac Surgery, The Fourth People's Hospital Affiliated to Tongji University, Hongkou District, Shanghai, China; 2 Department of Intensive Care Unit, The Fourth People's Hospital Affiliated to Tongji University, Hongkou District, Shanghai, China; 3 Department of Anesthesiology, Shanghai Jiangwan Hospital, Hongkou District, Shanghai, China

**Keywords:** color Doppler jet area, leaflet elasticity, mitral valve orifice area, postoperative mitral regurgitation, rheumatic mitral valve repair

## Abstract

**Background:**

Rheumatic mitral valve repair has been subject to clinical scrutiny due to the recurrence of postoperative regurgitation. This study aimed to identify factors associated with the development of mild or greater mitral regurgitation (MR) within 14 days after surgical repair.

**Methods:**

A total of 164 patients (mean age 44.73 years, 36 males) diagnosed with rheumatic mitral valve disease who underwent open-chest mitral valve repair at Shanghai Fourth People's Hospital, Tongji University School of Medicine, between March 17, 2023 and December 15, 2025, were enrolled. Mild MR was diagnosed using a comprehensive echocardiographic assessment, incorporating quantitative and semi-quantitative Doppler parameters—including color jet area (CJA) in the apical four-chamber view and vena contracta width (VCW), and the subjects were grouped accordingly.

**Results:**

A total of 164 patients were enrolled. Baseline clinical characteristics were well balanced between the two groups (all *P* > 0.05). At the 14-day postoperative time point, logistic regression analysis comparing the two groups revealed that preoperative CJA was significantly associated with non-minimal MR (*P* < 0.05) after adjustment for intraoperative concurrent stripping of thickened leaflets. However, this association became non-significant (*P* > 0.05) following additional adjustment for preoperative mitral valve orifice area (MVOA). Pearson correlation analysis revealed statistically significant associations between preoperative CJA and preoperative MVOA and between preoperative CJA and concurrent stripping of thickened leaflets (*P* < 0.05); variance inflation factor (VIF) values for both pairs were below 5 (VIF < 5), indicating absence of multicollinearity.

**Conclusion:**

Preoperative CJA may serve as a valuable adjunctive parameter for perioperative risk stratification and individualized surgical planning in rheumatic mitral valve repair surgery.

## 1 Introduction

Rheumatic heart disease represents a leading cause of cardiovascular morbidity and mortality worldwide. In 2019, the estimated national prevalence of rheumatic valvular disease in China was 9,073,096 cases (95% uncertainty interval: 7,393,886–10,994,319) (1). Rheumatic mitral valve disease typically presents with predominant MR during the acute phase (2). Over time, pathological involvement progresses from the commissures to involve the entire mitral valve apparatus—including leaflets, chordae tendineae, and papillary muscles—with severe structural alterations in subvalvular components and leaflet tissue (3–5) correlating strongly with progressive functional deterioration. Leaflet thickening and diminished tissue elasticity impair dynamic coaptation surface adjustment (6), while chordal thickening and calcification induce restrictive tethering, preventing adequate leaflet apposition—thereby establishing the anatomical substrate of rheumatic MR. Although mitral valve repair offers well-documented advantages such as preservation of native valve physiology (1, 7) and elimination of lifelong anticoagulation requirements (8), its broader adoption remains limited by persistently high rates of early failure and reoperation (9). Consequently, mitral valve replacement (MVR) accounts for over 95% of surgical interventions for rheumatic mitral disease globally. This study retrospectively enrolled patients who underwent open-chest mitral valve repair for rheumatic etiology at our institution, aiming to identify independent perioperative predictors of non-minimal MR (defined as ≥ mild MR) within 14 days postoperatively—thereby informing strategies to enhance procedural durability and long-term success.

## 2 Methods

### 2.1 Study population

This study was approved by the Ethics Committee of Shanghai Fourth People's Hospital, Tongji University School of Medicine (approval date: January 2, 2025; approval number: 2024245-002). As a retrospective observational study utilizing exclusively de-identified, anonymized clinical data with no capacity for subject re-identification, this research qualifies for waiver of informed consent in accordance with the institutional policies of the Ethics Committee. Patients diagnosed with rheumatic mitral valve disease at Shanghai Fourth People's Hospital Affiliated to Tongji University between March 17, 2023, and December 15, 2025, who underwent open mitral valve repair surgery at our institution, were enrolled in this study. To minimize inter-institutional variability in echocardiographic assessment and ensure data consistency, only patients who completed postoperative follow-up echocardiography at our center were included. A total of 164 patients with complete follow-up records were identified (baseline characteristics are summarized in Table 1).

**Table 1:** Baseline demographic and clinical characteristics of the study population.

Variables	Group S (*n* = 11)	Group M (*n* = 153)	*P*
Age (years)	53.00 (41.00, 61.00)	44.00 (37.00, 52.00)	0.06
Height (cm)	158.00 (153.00, 161.00)	160.00 (158.00, 166.50)	0.07
Weight (kg)	64.00 (49.00, 71.00)	60.00 (54.00, 66.00)	0.88
BMI	24.69 (21.63, 27.08)	22.96 (21.01, 24.90)	0.12
Sex: Male, *n* (%)	1.00 (9.10)	35.00 (22.88)	0.26
Hypertension, *n* (%)	0	8.00 (5.23)	1.00
History of coronary heart disease, *n* (%)	0	4.00 (2.61)	1.00
Smoking history, *n* (%)	0	16.00 (10.46)	0.60
Alcohol consumption history, *n* (%)	1.00 (9.10)	10.00 (6.54)	0.55
Blood glucose concentration (mmol/L)	4.40 (4.20, 4.80)	4.40 (4.00, 4.75)	0.58

Baseline demographic and clinical characteristics of the study population.

Group S, non-minimal MR/severe group (moderate/severe); Group M, mild MR group (none/trivial/mild).

### 2.2 Surgical procedure

Following standard induction of cardiopulmonary bypass, the left atrium was opened to allow direct visualization of the mitral valve apparatus and assessment of valvular pathology. The SCORE “4-step technique” was systematically performed as follows: (1) removal of fibrous plaques at the commissural regions; (2) approximation of the leaflets to evaluate the natural alignment of the commissures and determine the appropriate extent of commissurotomy; (3) division of fused commissures; and (4) release of the fused subvalvular apparatus, including incision of conjoined chordae tendineae and papillary muscles. Subsequently, additional thinning of the thickened anterior and posterior mitral leaflets was performed to restore leaflet mobility and flexibility. Valve competence was then assessed through saline distension testing (water test) and sizing with a valvuloplasty gauge, followed by implantation of an appropriately sized mitral annuloplasty ring. The atriotomy was closed in layers, completing the mitral valve repair procedure. After successful weaning from cardiopulmonary bypass and restoration of spontaneous cardiac rhythm, intraoperative transesophageal echocardiography (TEE) was performed to evaluate the immediate results. In cases where non-minimal MR (moderate or severe) regurgitation was detected, re-intervention was undertaken. When residual regurgitation was mild or less, the surgical procedure was considered satisfactory and the operation was concluded.

### 2.3 Statistical methods

Currently, the evaluation of MR remains primarily based on transthoracic echocardiography. According to established guidelines, MR is classified as mild when the following criteria are met—regardless of etiology: a small central jet, preserved leaflet morphology, VCW < 0.3 cm, absence of proximal flow convergence, and an A-wave—dominant mitral inflow pattern—thereby obviating the need for further quantitative assessment (10, 11). A small central jet is defined as a CJA < 4 cm² (12). The mitral valve orifice area was measured in the parasternal long-axis view at the level of the mitral valve and in the left ventricular short-axis view; transvalvular pressure gradients were assessed using continuous-wave Doppler in the apical four-chamber, two-chamber, or three-chamber views; VCW was measured in the apical four-chamber view; and CJA was quantified in the apical four-chamber, two-chamber, or three-chamber views during mid-systole. Current clinical practice guidelines (13) consistently recommend conservative management for patients with mild or asymptomatic (i.e., grade I or trace) MR, given their significantly more favorable long-term prognosis compared with those exhibiting moderate or severe MR. Repeat surgical intervention is not indicated in the absence of disease progression; reoperation is reserved exclusively for cases in which MR advances to meet established surgical indications—such as worsening symptoms, left ventricular dysfunction, or echocardiographic evidence of progressive remodeling. Consequently, the persistence of mild or no/minimal MR within the early postoperative period serves as a validated imaging biomarker reflecting successful anatomical and functional correction of the underlying valvular pathology. Mild MR was diagnosed according to established echocardiographic criteria—including CJA and VCW), and additional quantitative and qualitative parameters—and patients were stratified into two group accordingly. The mild MR group comprised patients who maintained mild or less regurgitation within 14 days postoperatively (none/trivial/mild) (hereafter, this cohort is designated Group M), whereas the non-minimal MR/severe group included those who developed moderate or greater mitral regurgitation during the same period (moderate/severe) (hereafter, this cohort is designated Group S). Predefined variables analyzed included preoperative MVOA, preoperative mitral CJA, history of percutaneous mitral balloon valvuloplasty, intraoperative mitral and tricuspid annuloplasty ring sizes, concurrent stripping of thickened leaflets, concomitant aortic valve repair, maze procedure, and left atrial appendage resection.

Data collection and entry were performed using EpiData 3.1 software, and data cleaning along with database management was carried out in Microsoft Excel. Statistical analyses were conducted using SPSS 29.0 software. Non-normally distributed continuous variables were summarized as medians and interquartile ranges (IQRs), while categorical variables were expressed as frequencies and proportions. Comparisons of normally distributed continuous variables between groups were performed using the independent samples Student's *t*-test, whereas the Mann–Whitney *U* test was applied for non-normally distributed variables. Categorical variables were compared using the chi-square test or Fisher's exact test, as appropriate. Univariate binary logistic regression analysis was used to evaluate the association between individual variables and the outcome of interest; variables with statistical significance in univariate analysis were further included in multivariate logistic regression models to identify independent predictors. A two-sided *P* value < 0.05 was considered statistically significant.

## 3 Results

(1) A total of 164 patients were enrolled in this study. Of these, 11 (6.71%) constituted the Group S and 153 (93.29%) Group M. Baseline characteristics were well balanced between groups: Group S comprised 1 male, with a median age of 53.0 years; In contrast, Group M included 35 males, with a median age of 44.0 years. No baseline variable differed significantly between groups (Table 1). (2) Statistically significant between-group differences were observed in preoperative CJA (median: 11.85 cm² vs. 4.20 cm², *P* = 0.01) and preoperative MVOA (median: 1.60 cm² vs. 1.10 cm², *P* = 0.01). No other baseline characteristics differed significantly between groups (all *P* > 0.05) (Table 2). (3) Univariate binary logistic regression identified intraoperative simultaneous stripping of thickened valve leaflets (OR = 4.41, 95% CI: 1.03–18.82, *P* = 0.045), preoperative CJA (OR = 1.17, 95% CI: 1.04–1.31, *P* = 0.01), and preoperative MVOA (OR = 3.33, 95% CI: 1.09–10.19, *P* = 0.04) as significant predictors of moderate/severe MR recurrence within 14 days postoperatively. No other variables demonstrated statistically significant associations (all *P* > 0.05). (4) In the multivariate logistic regression model adjusting for intraoperative simultaneous stripping of thickened mitral leaflets, preoperative CJA remained significantly associated with recurrence of moderate/severe MR within 14 days postoperatively (OR = 1.16; 95% CI: 1.03–1.30; *P* = 0.02). However, this association attenuated and became statistically non-significant after additional adjustment for preoperative MVOA (OR = 1.13; 95% CI: 0.97–1.31; *P* = 0.12). In the final model, no variable was independently associated with the outcome (all *P* > 0.05). Preoperative mitral valve CJA and MVOA were correlated (*r* = [0.445], *P* < 0.001), but not collinear (Tol. = 0.98, VIF = 1.02). Preoperative mitral valve CJA and concurrent stripping of thickened leaflets were correlated (*r* = [−0.218], *P* = 0.03), but not collinear (Tol. = 0.98, VIF = 1.02) (Tables 3–6).

**Table 2:** All variables included in the cohort, preoperative and during the operation.

Variables	Group S (*n* = 11)	Group M (*n* = 153)	*P*
Preoperative CJA (cm^2^)	11.85 (5.00, 15.10)	4.20 (2.70, 7.90)	0.01
History of closed mitral balloon valvuloplasty	1.00 (9.10)	16.00 (10.46)	1.00
Preoperative MVOA (cm^2^)	1.60 (1.18, 1.73)	1.10 (0.90, 1.40)	0.01
All variables included in the cohort, Preoperative.			

Variables	Group S (*n* = 11)	Group M (*n* = 153)	*P*
The size of the mitral valve annuloplasty ring implanted intraoperatively	33.82 ± 1.08	34.07 ± 0.98	0.48
The size of the tricuspid valve annuloplasty ring implanted intraoperatively	28.73 ± 2.41	28.39 ± 0.80	0.31
Concurrent stripping of thickened leaflets	8.00 (72.73)	141.00 (92.16)	0.07
AoGroup S: non-minimal MR/severe group (moderate/severe); Group M: mild MR group (none/trivial/mild); CJA, color jet area; MVOA, mitral valve orifice area.rtic valve plasty was performed concomitantly during the procedure	2.00 (18.18)	51.00 (33.33)	0.51
The Maze procedure was performed concomitantly during the operation	6.00 (54.55)	55.00 (35.95)	0.33
Concomitant resection of the left atrial appendage was performed during the operation	9.00 (81.82)	99.00 (64.71)	0.34

All variables included in the cohort, During the operation.

Group S, non-minimal MR/severe group (moderate/severe); Group M, mild MR group (none/trivial/mild); CJA, color jet area; MVOA, mitral valve orifice area.

**Table 3:** Univariate logistic regression analysis, preoperative.

Variables	OR	95% CI	*P*
History of closed mitral balloon valvuloplasty	1.17	0.14–9.73	0.89
Preoperative CJA (cm^2^)	1.17	1.04–1.31	0.01
Preoperative MVOA (cm^2^)	3.33	1.09–10.19	0.04
Univariate Logistic Regression Analysis, Preoperative.			
CJA, color jet area; MVOA, mitral valve orifice area.			

Variables	OR	95% CI	*P*
The size of the mitral valve annuloplasty ring implanted intraoperatively	0.78	0.42–1.44	0.42
The size of the tricuspid valve annuloplasty ring implanted intraoperatively	1.25	0.80–1.95	0.33
Concurrent stripping of thickened leaflets	4.41	1.03–18.82	0.045
Aortic valve plasty was performed concomitantly during the procedure	2.25	0.47–10.80	0.31
The Maze procedure was performed concomitantly during the operation	0.47	0.14–1.60	0.23
Concomitant resection of the left atrial appendage was performed during the operation	2.46	0.51–11.77	0.26

Univariate Logistic Regression Analysis, During the operation.

**Table 4:** Multivariate logistic regression analysis.

Variables	OR	95% CI	*P*
Concurrent stripping of thickened leaflets	2.27	0.36–14.39	0.39
Preoperative CJA (cm^2^)	1.16	1.03–1.30	0.02
Multivariate Logistic Regression Analysis.			
CJA, color jet area.			

Variables	OR	95% CI	*P*
Concurrent stripping of thickened leaflets	0.82	0.08–8.23	0.87
Preoperative CJA (cm^2^)	1.13	0.97–1.31	0.12
Preoperative MVOA (cm^2^)	1.25	0.30–5.20	0.76

Multivariate Logistic Regression Analysis.

CJA, color jet area; MVOA, mitral valve orifice area.

**Table 5:** Pearson correlation analysis.

Variables		Preoperative MVOA (cm^2^)	Mean mitral transvalvular pressure gradient (mmHg)	Concurrent stripping of thickened leaflets
Preoperative CJA (cm^2^)	*r*	0.445	−0.10	−0.218
	*P*	0.00	0.31	0.03

Pearson correlation analysis.

CJA, color jet area; MVOA, mitral valve orifice area.

**Table 6:** Collinearity analysis.

Variables	*P*	Tolerance	VIF
Preoperative MVOA (cm^2^)	0.00	0.98	1.02
Concurrent stripping of thickened leaflets	0.29	0.98	1.02

Collinearity analysis.

Dependent variable: Preoperative CJA.

CJA, color jet area; MVOA, mitral valve orifice area.

## 4 Discussion

This study investigated predictors of moderate/severe MR recurrence within 14 days following rheumatic mitral valve repair and identified three key findings: (1) Despite baseline comparability between the two patient groups, our results diverged from prior evidence regarding several procedural variables—including prior mitral balloon valvuloplasty (14), intraoperative mitral and tricuspid annuloplasty ring size, concomitant aortic valve repair (15), maze procedure (15), and left atrial appendage excision (16, 17). These factors likely exert their influence on MR recurrence indirectly—through modulation of ventricular remodeling pathways—rather than via direct effects on mitral valve coaptation or leaflet mechanics. However, because the enrolled cohort had not yet undergone established compensatory ventricular remodeling, this indirect pathway remained inactive and thus undetectable within the observed 14-day postoperative window; (2) Concurrent stripping of thickened leaflets was associated with an increased risk of moderate/severe regurgitation recurrence within 14 days postoperatively, and this association was partially mediated by the preoperative CJA. (3) Preoperative mitral CJA and MVOA exhibited a statistical suppression effect in multivariable modeling. Both parameters quantitatively capture a shared underlying pathological feature of rheumatic mitral valve disease—valvular structural stiffness—and collectively represent the principal anatomical–functional determinant of early post-repair regurgitation recurrence.

Although leaflet thinning is theoretically capable of restoring coaptation competence (18, 19), however, due to two fundamental technical limitations inherent to current surgical practice—namely. Firstly, during intraoperative resection of thickened mitral leaflets, the rough band on the ventricular surface that anchors the chordae tendineae (20) remains inaccessible for surgical modulation. This region exhibits marked surface very rough and may disrupt physiological force transmission along the chordal apparatus, predisposing to chordal rupture. Consequently, coexisting zones of preserved flexibility and pathological rigidity—emerge within a single leaflet. Leaflet flexibility is essential for uniform stress distribution across its surface (21); loss of this property in locally fibrotic or calcified segments compromises biomechanical homeostasis, leading to focal stress concentration, iatrogenic chordal injury, and subsequent secondary MR. At the same time, the anatomical substrate essential for coaptation reserve is localized predominantly in the region subjacent to the coaptation line.; if mobility in this critical zone remains impaired, global coaptation improvement will be inherently limited. Collectively, the “leaflet stripping” strategy is limited by the inherent constraints of current surgical technology.; thus, the recurrence of MR will be manifested by the CJA. Second, histopathological analysis revealed that the boundary between the rheumatic mitral valve leaflet lesion tissue and the adjacent normal tissue was indistinct (22). Consequently, intraoperative visual assessment cannot reliably delineate the precise boundary for safe dissection (30), and current instrumentation lacks the selectivity to isolate pathological matrix without compromising native collagen architecture. Forced or overly aggressive stripping frequently results in microtears within the lamina fibrosa or endothelial denudation—both of which compromise local tensile strength. Following surgical intervention, elevated systolic ventricular pressure induces heterogeneous strain distribution across the mitral leaflet due to regional variations in tissue compliance—specifically, the juxtaposition of pliable and stiffened segments—resulting in disproportionate bulging of mechanically compromised zones, pulling the relatively rigid segment immediately below the coaptation line toward the annulus and paradoxically widening the coaptation gap. While thinning it, the area of the valve orifice is simultaneously artificially enlarged, compromises the orifice's intrinsic compensatory capacity (a narrowed mitral orifice reduces the coaptation distance, thereby facilitating earlier leaflet apposition during systole—a intrinsic biomechanical compensation mechanism),—a plausible structural contributor to early post-repair MR. The recurrence of MR will be manifested by the CJA too. The clinical effect in patients with not very severe thickening of the leaflets lmay fall short of the theoretical objective of “fully restoring global leaflet mobility”, particular surgical caution is warranted in these patients intraoperatively. Finally, it is plausible that the myocardial and valvular remodeling triggered by leaflet thinning unfolds over a timeframe extending well beyond 14 days; definitive assessment of its long-term functional consequences therefore necessitates extended follow-up beyond the early postoperative window. Should future advances overcome the current technical limitations—and extend longitudinal follow-up beyond the completion of cardiac remodeling—the leaflet-thinning intervention may robustly manifest its therapeutic potential, including enhanced valve compliance, and favorable modulation of ventricular remodeling.

The degree of structural stiffness in rheumatic mitral valves is a key determinant of postoperative MR recurrence (18). Prior evidence indicates that preoperative mitral stenosis compromises the durability and functional outcomes of rheumatic mitral valve repair (23); critically, this reflects an underlying, irreversible fibrocalcific stiffening of the leaflets and subvalvular apparatus. Our findings corroborate this pathophysiological link. Yet, a major unmet need remains: the lack of a comprehensive, objective, and clinically applicable metric to quantify mitral valve structural stiffness. The widely adopted Wilkins score—designed exclusively for patient selection and procedural outcome assessment in percutaneous balloon mitral valvuloplasty for mitral stenosis (24)—lacks sensitivity to regurgitant hemodynamics and is therefore unsuitable for rheumatic patients with predominant MR, mixed disease. In contrast, this study demonstrates that the CJA serves as a robust, reproducible, and quantifiable imaging-derived surrogate for intrinsic valve stiffness. Importantly, CJA can predict perioperative risk and informs tailored surgical decision-making. It should be emphasized that the CJA itself is not a pathogenic factor. Its clinical risk correlation stems from the irreversible stiffness of the valve structure, which it represents.

To systematically define the clinical decision-support value of the CJA, this study stratifies rheumatic mitral valve disease into three pathophysiologically distinct stages—termed the *MR progression phase*, the *MR attenuation phase*, and the *MR re enlargement phase*—based on longitudinal hemodynamic and structural evolution. The MR progression phase corresponds to early rheumatic involvement, characterized predominantly by MR. Progressive MR lead to increasing MVOA, which further compromises coaptation surface area and amplifies regurgitant volume (25). Clinically, both CJA and MVOA increase in parallel during this phase. Crucially, valvular structural stiffness remains minimal; consequently, repair success rates are exceptionally high, surgeons with documented experience in rheumatic mitral reconstruction are qualified to perform valve repair surgery. As disease advances, patients enter the MR attenuation phase. Here, CJA declines—not due to milder pathology or preserved tissue compliance, but rather as a consequence of progressive fibrocalcific orifice narrowing that mechanically restricts regurgitant jet expansion (26). Accordingly, both CJA and MVOA decrease concurrently. However, this apparent “improvement” in hemodynamics masks progressive subvalvular and leaflet stiffening. Therefore, surgeons performing rheumatic mitral repair must possess substantial, dedicated expertise in this specific domain; independent surgical intervention is contraindicated for those lacking documented proficiency or formal, structured training in rheumatic valve reconstruction; In the MR re enlargement phase, multisite irreversible stiffening results in near-complete loss of leaflet mobility and coaptation capacity. Paradoxically, despite marked reduction in MVOA, CJA increases abnormally due to severe leaflet shortening, contracture, and fixed malpositioning (27), often exceeding baseline values observed in the MR progression phase. Rheumatic mitral valve repair aims fundamentally to restore physiological hemodynamics through structural correction. Greater lesion complexity is strongly associated with an increased incidence of intraoperative residual structural abnormalities. Over time, these uncorrected elements progressively impair the reconstructed valvular apparatus, disrupting coaptation geometry and flow dynamics. This leads to composite valvular dysfunction and confers a substantial risk of late recurrence of either stenosis or regurgitation (28, 29). To optimize long-term outcomes, intraoperative assessment must be comprehensive and systematic—evaluating all components of the mitral apparatus. Achieving this objective demands exceptional surgical expertise and deep, rheumatic-specific clinical experience. Consequently, mitral valve repair should be performed exclusively by high-volume surgical teams with sustained, dedicated expertise in rheumatic mitral reconstruction. In contrast, for surgeons lacking such proficiency, mitral valve replacement—not attempted repair—represents the evidence-based, patient-centered standard of care. Findings from the three-phase analysis collectively demonstrate that CJA and MVOA capture distinct, complementary manifestations of valvular structural stiffness—CJA reflects the hemodynamic functional consequences of valvular structural stiffness, whereas MVOA captures its anatomical-morphological correlates—these two parameters are mechanistically distinct and non-redundant. Future work should validate these staging criteria in large, multicenter prospective cohorts to rigorously establish the causal association between CJA-defined structural stiffness and the risk of moderate/severe MR recurrence within 14 days postoperatively—thereby enabling precise quantification of its predictive performance and real-world clinical utility.

## 5 Limitation

(1) This study has a relatively limited sample size, with only 164 patients enrolled, classifying it as a single-center, small-sample investigation. While this may constrain the statistical power and generalizability of the findings, future expansion of the cohort through prolonged follow-up and inclusion of additional patients is anticipated. Furthermore, the limitation can be addressed by adopting a multicenter collaborative design in subsequent studies to enhance external validity. (2) This study is a recently conducted clinical observational study. While perioperative confounding factors—including those related to surgical technique, timing, and early postoperative management—were explicitly accounted for during the initial 14-day postoperative period, the overall follow-up duration remains limited. This constraint may attenuate the statistical power and limit both the internal validity of associations identified and the external validity (i.e., generalizability) of the findings. To strengthen analytical rigor, we applied stringent variable selection criteria and excluded patients with substantial missing clinical or echocardiographic data, thereby mitigating potential biases—including information bias and residual confounding—that could compromise estimation accuracy. Future investigations will implement extended longitudinal follow-up to robustly assess long-term clinical outcomes, disease progression, and the durability of therapeutic effects. (3) A key methodological limitation warrants acknowledgment: the majority of patients in this cohort underwent concomitant tricuspid valve annuloplasty during mitral valve surgery. As a result, postoperative tricuspid regurgitation was markedly attenuated—predominantly absent or trace—thereby precluding reliable noninvasive estimation of PASP via continuous-wave Doppler interrogation of the tricuspid regurgitant jet on transthoracic echocardiography. This constraint limits the accuracy and generalizability of pulmonary hemodynamic assessment in the present study. To address this, future investigations should incorporate invasive hemodynamic measurement—specifically, right heart catheterization—to obtain direct, quantitative PASP values and enable more robust characterization of pulmonary vascular status. (4) We emphasize that while mitral valve morphological remodeling is the principal driver of disease progression in this cohort, CJA serves only as an indirect, static morphometric proxy for irreversible fibrocalcific stiffening. Its use as a standalone quantitative metric for MR severity entails inherent constraints: measurements are sensitive to loading conditions, transducer positioning, image resolution, and interobserver variability—factors introducing potential systematic bias. Notably, current guideline-defined criteria for mild or trace MR are intentionally permissive, requiring fewer echocardiographic confirmatory findings. In our cohort, alternative quantitative MR parameters—including VCW—exhibited widespread non-measurability (e.g., 121 missing VCW values), primarily due to their inherently low magnitude in low-grade regurgitation—a well-documented echocardiographic phenomenon—not data artifact. These parameters were therefore excluded from statistical modeling. The next step is to develop and rigorously validate a novel, integrated index—grounded in established imaging modalities and functional scoring systems—that objectively quantifies mitral valve structural stiffness.

## 6 Conclusions

A significantly elevated CJA, particularly when accompanied by a significantly reduced MVOA, may indicate extensive rheumatic involvement and pronounced structural rigidity of both the mitral valve leaflets and subvalvular apparatus. These features confer substantial technical complexity to mitral valve repair and are associated with an elevated risk of early repair failure. Consequently, such cases should be managed exclusively at high-volume centers with documented expertise in rheumatic mitral valve reconstruction; performance by institutions without established proficiency in this specialized technique is strongly discouraged.

## Data Availability

The original contributions presented in the study are included in the article/Supplementary Material, further inquiries can be directed to the corresponding authors.
