# Correction: Factors associated with recurrence of mitral regurgitation 14 days after rheumatic mitral valve repair

**DOI:** 10.3389/fcvm.2026.1893147

**Published:** 2026-06-16

**Authors:** RenDong Liang, Ding Zhang, QingYu Sun, BiJun Zhao, Xu Meng

**Affiliations:** 1 Department of Cardiac Surgery, The Fourth People's Hospital Affiliated to Tongji University, Hongkou District, Shanghai, China; 2 Department of Intensive Care Unit, The Fourth People's Hospital Affiliated to Tongji University, Hongkou District, Shanghai, China; 3 Department of Anesthesiology, Shanghai Jiangwan Hospital, Hongkou District, Shanghai, China

**Keywords:** color Doppler jet area, leaflet elasticity, mitral valve orifice area, postoperative mitral regurgitation, rheumatic mitral valve repair

In the published article, there was an omission regarding the use of generative AI. The first sentence in the Generative AI statement was displayed as “*The author(s) declared that generative AI was not used in the creation of this manuscript.*” The correct version is “*The author(s) declared that generative AI was used in the creation of this manuscript.* During the preparation of this work, the author(s) used Youdao's web-based AI translation service（Ziyue Translation LLM 2.0） solely for English translation and linguistic polishing of the text. After using this tool, the author(s) reviewed and edited the content as needed and take(s) full responsibility for the content of the publication.’’

The original version of this article has been updated.

